# Association of Social and Behavioral Risk Factors With Mortality Among US Veterans With COVID-19

**DOI:** 10.1001/jamanetworkopen.2021.13031

**Published:** 2021-06-09

**Authors:** J. Daniel Kelly, Dawn M. Bravata, Stephen Bent, Charlie M. Wray, Samuel J. Leonard, W. John Boscardin, Laura J. Myers, Salomeh Keyhani

**Affiliations:** 1 San Francisco VA Medical Center, San Francisco, California; 2 Department of Epidemiology and Biostatistics, University of California, San Francisco, San Francisco; 3 Institute for Global Health Sciences, University of California, San Francisco, San Francisco; 4 F.I. Proctor Foundation, University of California, San Francisco, San Francisco; 5 US Department of Veterans Affairs, Health Services and Development, Center for Health Information and Communication, Indianapolis, Indiana; 6 Department of Medicine, Richard L. Roudebush VA Medical Center, Indianapolis, Indiana; 7 Veterans Affairs Medical Center, Indianapolis, Indiana; 8 Department of Medicine, Indiana University School of Medicine, Indianapolis; 9 Regenstrief Institute, Indianapolis, Indiana; 10 Department of Medicine, University of California, San Francisco, San Francisco

## Abstract

**Importance:**

The US Department of Veterans Affairs (VA) offers programs that reduce barriers to care for veterans and those with housing instability, poverty, and substance use disorder. In this setting, however, the role that social and behavioral risk factors play in COVID-19 outcomes is unclear.

**Objective:**

To examine whether social and behavioral risk factors were associated with mortality among US veterans with COVID-19 and whether this association might be modified by race/ethnicity.

**Design, Setting, and Participants:**

This cohort study obtained data from the VA Corporate Data Warehouse to form a cohort of veterans who received a positive COVID-19 test result between March 2 and September 30, 2020, in a VA health care facility. All veterans who met the inclusion criteria were eligible to participate in the study, and participants were followed up for 30 days after the first SARS-CoV-2 or COVID-19 diagnosis. The final follow-up date was October 31, 2020.

**Exposures:**

Social risk factors included housing problems and financial hardship. Behavioral risk factors included current tobacco use, alcohol use, and substance use.

**Main Outcomes and Measures:**

The primary outcome was all-cause mortality in the 30-day period after the SARS-CoV-2 or COVID-19 diagnosis date. Multivariable logistic regression was used to estimate odds ratios, clustering for health care facilities and adjusting for age, sex, race, ethnicity, marital status, clinical factors, and month of COVID-19 diagnosis.

**Results:**

Among 27 640 veterans with COVID-19 who were included in the analysis, 24 496 were men (88.6%) and the mean (SD) age was 57.2 (16.6) years. A total of 3090 veterans (11.2%) had housing problems, 4450 (16.1%) had financial hardship, 5358 (19.4%) used alcohol, and 3569 (12.9%) reported substance use. Hospitalization occurred in 7663 veterans (27.7%), and 1230 veterans (4.5%) died. Housing problems (adjusted odds ratio [AOR], 0.96; 95% CI, 0.77-1.19; *P* = .70), financial hardship (AOR, 1.13; 95% CI, 0.97-1.31; *P* = .11), alcohol use (AOR, 0.82; 95% CI, 0.68-1.01; *P* = .06), current tobacco use (AOR, 0.85; 95% CI, 0.68-1.06; *P* = .14), and substance use (AOR, 0.90; 95% CI, 0.71-1.15; *P* = .41) were not associated with higher mortality. Interaction analyses by race/ethnicity did not find associations between mortality and social and behavioral risk factors.

**Conclusions and Relevance:**

Results of this study showed that, in an integrated health system such as the VA, social and behavioral risk factors were not associated with mortality from COVID-19. Further research is needed to substantiate the potential of an integrated health system to be a model of support services for households with COVID-19 and populations who are at risk for the disease.

## Key Points

**Question:**

Are social and behavioral risk factors associated with mortality in US veterans with COVID-19?

**Findings:**

In this cohort study of 27 640 veterans who received a positive test result for COVID-19, risk factors such as housing problems, financial hardship, alcohol use, tobacco use, and substance use were not associated with higher mortality.

**Meaning:**

This study found no association between social and behavioral risk factors and death from COVID-19 in an integrated VA health system; such a system is known to transcend social vulnerabilities and has the potential to be a model of support services for households and at-risk populations in the US.

## Teaser

This cohort study examines the implications of inadequate housing; financial difficulty; and alcohol, tobacco, or substance use as well as race/ethnicity and other covariates for veterans with symptomatic SARS-CoV-2 infection or COVID-19.

## Introduction

The COVID-19 pandemic has exposed health disparities in the United States.^1^ In retracing the origins of the disease in the US, studies have found that the SARS-CoV-2 virus was introduced by individuals traveling around the world^2^ and that transmission chains eventually reached poor, vulnerable, and marginalized communities, which have been disproportionately affected and sustained community transmission.^3^ Although the pandemic has affected all lives in some way, it has also highlighted the health disparities in the US.^4^ Many people with low incomes are unable to work remotely because of their jobs as essential workers or to practice social distancing because of population density, making exposures to COVID-19 as well as transmission within families and social networks unavoidable.^5,6^ This situation has been associated with a substantial imbalance in case prevalence: socially vulnerable populations tend to have limited access to health care, less social support, and greater comorbidities, all of which contribute to worse outcomes and higher mortality from COVID-19.^7,8,9,10^

Social and behavioral risk factors have been found to be associated with COVID-19 outcomes.^11,12,13,14^ In poor, vulnerable, and marginalized communities, the prevalence of tobacco, alcohol, and drug use is high, and the various substances can be independent risk factors in COVID-19 outcomes.^15^ Veterans living throughout the US have diverse socioeconomic and behavioral backgrounds as well as diverse races/ethnicities.^16,17^ Although COVID-19 studies have increasingly reported that mortality is associated with race/ethnicity, US Department of Veterans Affairs (VA)–specific studies have shown no difference in mortality by race/ethnicity.^18,19^ The VA health system offers programs that are intended to reduce barriers to care for veterans as well as those with housing instability, poverty, and substance use disorder^20,21,22,23^; in this setting, it is unclear whether social and behavioral risk factors are associated with poor COVID-19 outcomes.

Juxtaposed with the structural inequity documented in the general US health care system is the structural equality cultivated within the VA system, with evidence of few racial disparities in health outcomes.^24,25,26^ The VA Office of Health Equity creates quality assurance metrics with health care teams to identify health inequities (eg, food insecurity); track them in the annual National Veteran Health Equity Report; and eliminate them through several personalized strategies, including tailoring outreach, earning trust, adapting treatment, and forming community partnerships.^27^

In this study, we used data from the VA to examine whether social and behavioral risk factors (housing problems; financial hardship; and tobacco, alcohol, and substance use) were associated with mortality among US veterans with COVID-19 and whether this association might be modified by race/ethnicity. We hypothesized that among veterans diagnosed with COVID-19, social and behavioral risk factors were not associated with mortality, even when the association was modified by race/ethnicity.

## Methods

The institutional review board of the University of California, San Francisco approved this cohort study and waived the need for informed consent because the research involved no more than minimal risk to participants. Data were collected from March 2 to September 31, 2020. We followed the Strengthening the Reporting of Observational Studies in Epidemiology (STROBE) reporting guideline.

### Study Design, Setting, Participants, and Procedures

Using the VA Corporate Data Warehouse,^28^ we designed a cohort study of veterans who were diagnosed with COVID-19. The VA is a national health system for US veterans with localized services, including COVID-19 laboratory testing and reporting. The robust electronic health record system of the VA allows for a well-characterized cohort with mortality estimates that are updated on a quarterly basis. March 2, 2020, marked the earliest time point that a positive polymerase chain reaction test result for SARS-CoV-2 was seen in the VA laboratory system.

We assessed the eligibility of 38 504 individuals who received their first test result of RNA positivity for SARS-CoV-2 between March 2 and September 30, 2020, in a VA health care facility.^28^ We excluded 6394 nonveteran employees and 1124 veterans who did not receive VA primary care within 2 years before their SARS-CoV-2 test result to ensure that the study cohort included only those who obtained care in the VA. In addition, we did not include veterans who were tested outside of the VA because we may not have had access to all of their clinical information. By including only veterans who received primary care and were tested in the VA, we minimized missing baseline data. Because our focus was on identifying a veteran population living in the community, we excluded 2401 veterans who received hospice or palliative care within 1 year of the test result and those who were residents of a community living center (eg, VA nursing home). We also excluded 945 veterans with a positive COVID-19 test result after surveillance or screening before a procedure, as indicated by laboratory codes, to ensure that the study population was symptomatic.

All individuals who met the inclusion criteria were eligible to participate in the study and formed the analysis cohort. Veterans were followed up for 30 days after their first SARS-CoV-2 or COVID-19 diagnosis. September 30, 2020, was the end date of new diagnoses because VA mortality data are updated on a quarterly basis.

### Data Sources and Measurements

All data on exposure, outcome, and covariate measurements were retrieved from the VA Corporate Data Warehouse.^29^ We used a combination of *International Statistical Classification of Diseases and Related Health Problems, Tenth Revision* (*ICD-10*), procedure, outpatient diagnosis, and laboratory codes to extract these variables. We reviewed a random sample of 50 medical records to verify the capture of symptomatic individuals in the data set given that asymptomatic individuals by definition have zero probability of COVID-19–related mortality. Of the 50 medical records, we could not determine symptom status in 1 medical record, and no symptoms were reported for 1 medical record; the other 48 medical records identified symptoms among all veterans. For select variables (as described in the following paragraph), we used other sources of data. eTable 1 in the Supplement provides details on how each variable was constructed.

The exposure variables were social and behavioral risk factors. Social risk factors were defined as housing problems and financial hardship. We used a combination of *ICD-10* codes on housing instability and receipt of VA housing services to identify veterans with evidence of housing problems. We identified an individual as having financial hardship according to *ICD-10* codes that suggested poverty or financial hardship as well as the VA priority group. Income level, VA pension benefits, and receipt of Medicaid are considered in the assignment of veterans to priority groups. The VA adjusts annual income levels on the basis of resident zip code in consideration of priority groups, offering a measurement of financial hardship.^30^ Behavioral risk factors were defined as tobacco, alcohol, and substance use. Current tobacco use (eg, smoking within the past 1 year) was identified with an existing VA algorithm that was updated to include smoking-related *ICD-10* diagnosis and clinic stop codes.^31^ Alcohol use was identified with a combination of *ICD-10* codes and Alcohol Use Disorders Identification Test (Audit-C) scores.^32^ Substance use was identified according to *ICD-10* codes. These *ICD-10* codes were considered during the 2 years before the study period. We ascertained the presence or absence of a social and behavioral risk factor on the basis of a participant having at least 1 *ICD-10* code during this 2-year period.

We reviewed a random sample of 25 medical records for each of the following variables: housing problems, current tobacco use, alcohol use, and substance use (a total of 100 records). We confirmed through medical record review that each social and behavioral risk factor identified using *ICD-10* codes and other data sources was present in the 2 years before the index COVID-19 diagnosis. We did not conduct medical records review on the financial hardship variable because that was largely designated by the VA priority score after the VA conducted an in-depth assessment of veterans when they enrolled to receive care in the VA health system.

### Primary Outcome and Covariates

The primary outcome was all-cause mortality in the 30-day period after the first test date of laboratory-confirmed SARS-CoV-2 infection or COVID-19 in a veteran. Mortality data were collected from the VA Corporate Data Warehouse and the VA Vital Status Files.^29^ When these available sources are combined, the resulting mortality data are comparable to the National Death Index in both accuracy and completeness. For the VA users, the combined data were 100% complete and 97.9% accurate to the date compared with the National Death Index.^33^ The final follow-up date was October 31, 2020. We considered other independent factors of mortality among veterans with COVID-19 and selected the following covariates: age, sex, race, ethnicity, marital status, comorbid conditions, body mass index, month of COVID-19 diagnosis, and hospitalization within 30 days. Race/ethnicity was self-reported by the individual, defined by the VA as a quality assurance mechanism for researchers, and assessed in the study because of the focus on health disparities.^34^ Hospitalization was identified using codes for inpatient admission (fee basis or VA) within 30 days of the first positive polymerase chain reaction test result for SARS-CoV-2 or COVID-19; otherwise, these covariates were baseline data that were collected before the test date.

Comorbid conditions included hypertension, ischemic heart disease, atrial fibrillation, stroke, diabetes or insulin use, heart failure, chronic kidney disease, chronic obstructive pulmonary disease or bronchiectasis, asthma, pneumonia, sleep apnea, deep venous thrombosis or pulmonary embolism, rheumatoid arthritis or other inflammatory conditions, cancer, HIV/AIDS, dementia, cirrhosis or hepatitis, home oxygen use in the past year, and mental health condition (depression, bipolar disorder, psychosis, and anxiety disorders). We used at least 1 inpatient *ICD-10* code or 2 outpatient diagnosis codes in the 2 years before the test date to identify the presence or absence of comorbidities.^35^

### Statistical Analysis

We performed descriptive analyses to estimate the prevalence of the covariates in the cohort. We assessed the prevalence of each social and behavioral risk factor by race/ethnicity, and we performed heterogeneity tests. Then, we assessed the association of social and behavioral risk factors with mortality. We fit multivariable logistic regression models, accounting for clustering through the addition of random effects for each VA health care facility, to estimate unadjusted and adjusted odds ratios (ORs). These assessments included iterative model building to understand the role of covariates (age, sex, race, ethnicity, marital status, clinical factors, health care facility, month of COVID-19 diagnosis) (eTable 2 in the Supplement). Clinical factors included body mass index and all comorbid conditions. These estimates were considered to be statistically significant if the CIs of the ORs did not cross the null value.

We tested the social and behavioral risk factors for interaction if race/ethnicity was associated with mortality. To this end, we took the product of each social and behavioral risk factor and race/ethnicity to create interaction terms. We assessed each possible interaction with a series of generalized linear models in which each model included the factor of interest (eg, housing problems) and its interaction term. Findings were considered to be statistically significant at a 2-sided *P* < .10 and if the factor was clinically relevant (eg, American Indian or Alaska Native vs White veterans). We used R, version 1.2.5019, including the glmm package (R Foundation for Statistical Computing), to conduct all analyses.

## Results

The analysis cohort consisted of 27 640 veterans (Figure). Of these veterans, the mean (SD) age was 57.2 (16.6) years, and 24 496 were male (88.6%) and 3144 were female (11.4%) participants. Black (9745 [35.3%]) and Hispanic (3896 [14.1%]) veterans were well represented, and hypertension (14 693 [53.2%]) was the most common comorbid condition. The proportion of veterans with inadequate housing (3090 [11.2%]) and financial hardship (4450 [16.1%]) was comparable to the US poverty rate of 10.5% in 2019.^36^ Alcohol use was the most prevalent behavioral risk factor (5358 [19.4%]), but current tobacco use (4910 [17.8%]) and substance use (3569 [12.9%]) were also relatively common. We observed high levels of hospitalization (7663 [27.7%]) and death (1230 [4.5%]) (Table 1).

**Figure.**
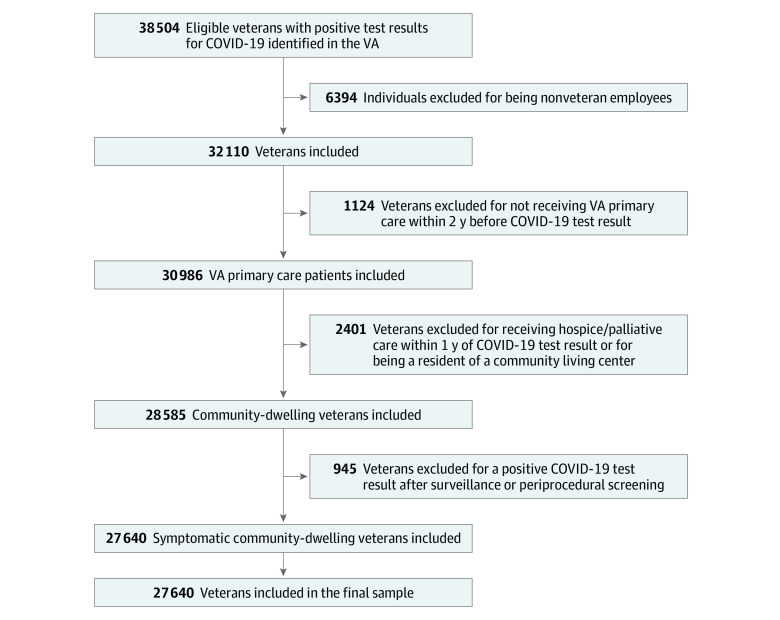
Flow Diagram of COVID-19 Cohort VA indicates US Department of Veterans Affairs.

**Table 1.** Characteristics of the US Veteran Cohort With COVID-19

Characteristic	No. (%)
No.	27 640
Age, y	
<55	11 444 (41.4)
55-59	2811 (10.2)
60-64	3197 (11.6)
65-69	2791 (10.1)
70-74	3869 (14.0)
75-79	1651 (6.0)
≥80	1877 (6.8)
Sex	
Female	3144 (11.4)
Male	24 496 (88.6)
Race	
White	15 113 (54.7)
Black	9745 (35.3)
Asian	328 (1.2)
American Indian or Alaska Native	283 (1.0)
Native Hawaiian or other Pacific Islander	316 (1.1)
Unknown	1855 (6.7)
Ethnicity	
Hispanic	3896 (14.1)
Not Hispanic	22 846 (82.7)
Unknown	894 (3.2)
Marital status	
Married	12 583 (45.5)
Social risk factors	
Inadequate housing	3090 (11.2)
Financial hardship	4450 (16.1)
No social risk factors	21 151 (76.5)
Behavioral risk factors	
Current tobacco use	4910 (17.8)
Alcohol use	5358 (19.4)
Substance use	3569 (12.9)
No behavioral risk factors	18 492 (66.9)
Comorbid conditions	
Hypertension	14 693 (53.2)
Ischemic heart disease, cardiac disease intervention, or myocardial infarction	3989 (14.4)
Atrial fibrillation	2054 (7.4)
Stroke	757 (2.7)
Diabetes or insulin use	8572 (31.0)
Congestive heart failure	2184 (7.9)
Chronic kidney disease	7233 (26.2)
Dialysis	426 (1.5)
COPD or bronchiectasis	3050 (11.0)
Asthma	1487 (5.4)
Pneumonia	893 (3.2)
Sleep apnea	4872 (17.6)
Deep venous thrombus or pulmonary embolism	672 (2.4)
Rheumatoid arthritis or other inflammatory conditions	590 (2.1)
Cancer, including prostate cancer	2080 (7.5)
HIV and AIDS	332 (1.2)
Dementia	1037 (3.8)
Cirrhosis or hepatitis	2014 (7.3)
Home oxygen in past year	818 (3.0)
No clinical conditions	7318 (26.5)
Mental health conditions	
Depression	7862 (28.4)
Bipolar disorder	931 (3.4)
Psychosis	885 (3.2)
Anxiety disorders	4682 (16.9)
No mental health conditions	17 470 (63.2)
BMI	
<18.5	119 (0.4)
18.5-24.9	3369 (12.2)
25.0-29.9	8110 (29.3)
≥30	13 717 (49.6)
Unknown	2325 (8.4)
VA region	
Northeast	7454 (27.0)
Southeast	10 889 (39.4)
Continental	4905 (17.7)
Pacific	4392 (15.9)
Mortality and hospitalization	
Died within 30 d	1230 (4.5)
Hospitalized within 30 d	7663 (27.7)
Month of COVID-19 diagnosis	
March	1885 (6.8)
April	2941 (10.6)
May	1745 (6.3)
June	4061 (14.7)
July	8623 (31.2)
August	4494 (16.3)
September	3891 (14.1)

Abbreviations: BMI, body mass index (calculated as weight in kilograms divided by height in meters squared); COPD, chronic obstructive pulmonary disease; VA, US Department of Veterans Affairs.

Of the social and behavioral risk factors, housing problems (adjusted OR [AOR], 0.96; 95% CI, 0.77-1.19; *P* = .70), financial hardship (AOR, 1.13; 95% CI, 0.97-1.31; *P* = .11), current tobacco use (AOR, 0.85; 95% CI, 0.68-1.06; *P* = .14), alcohol use (AOR, 0.82; 95% CI, 0.68-1.01; *P* = .06), and substance use (AOR, 0.90; 95% CI, 0.71-1.15; *P* = .41) did not have statistically significant associations with mortality. Other factors in the model had statistically significant associations, including older age (eg, AOR, 75-79 years: 16.04; 95% CI, 11.24-22.91; *P* < .001), Asian (AOR, 2.02; 95% CI, 1.06-3.86; *P* = .03) and American Indian or Alaska Native (AOR, 2.41; 95% CI, 1.45-4.02; *P* = .001) races, diabetes (AOR, 1.40; 95% CI, 1.22-1.60; *P* < .001), chronic kidney disease (AOR, 1.82; 95% CI, 1.60-2.08; *P* < .001), dementia (AOR, 2.08; 95% CI, 1.73-2.51; *P* < .001), cirrhosis or hepatitis (AOR, 1.30; 95% CI, 1.05-1.61; *P* = .02), and home oxygen use in the past year (AOR, 1.38; 95% CI, 1.07-1.79; *P* = .01) (Table 2).

**Table 2.** Associations of Social and Behavioral Risk Factors With 30-Day Mortality Among US Veterans With COVID-19^a^

Variable	OR	*P* value	Adjusted OR	*P* value
Social and behavioral risk factors				
Housing problems	0.86 (0.71-1.04)	.13	0.96 (0.77-1.19)	.70
Financial hardship	1.86 (1.63-2.12)	<.001	1.13 (0.97-1.31)	.11
Current tobacco use	0.63 (0.53-0.75)	<.001	0.85 (0.68-1.06)	.14
Alcohol use	0.53 (0.44-0.63)	<.001	0.82 (0.68-1.01)	.06
Substance use	0.83 (0.69-0.99)	.04	0.90 (0.71-1.15)	.41
Age, y				
<55	1 [Reference]		1 [Reference]	
55-59	0.30 (0.22-0.41)	<.001	2.75 (1.8-4.21)	<.001
60-64	0.67 (0.54-0.82)	<.001	5.12 (3.56-7.35)	<.001
65-69	1.04 (0.86-1.25)	.71	7.06 (4.94-10.08)	<.001
70-74	2.23 (1.96-2.55)	<.001	12.37 (8.86-17.29)	<.001
75-79	3.10 (2.63-3.65)	<.001	16.04 (11.24-22.91)	<.001
≥80	8.39 (7.37-9.55)	<.001	30.78 (21.74-43.57)	<.001
Sex				
Female	1 [Reference]		1 [Reference]	
Male	4.42 (3.17-6.18)	<.001	1.31 (0.91-1.87)	.15
Race				
White	1 [Reference]		1 [Reference]	
Black	1.09 (0.97-1.22)	.17	1.10 (0.95-1.28)	.20
Asian	0.81 (0.46-1.45)	.48	2.02 (1.06-3.86)	.03
American Indian or Alaska Native	1.83 (1.18-2.83)	.01	2.41 (1.45-4.02)	.001
Native Hawaiian or other Pacific Islander	0.68 (0.32-1.22)	.17	0.97 (0.48-1.99)	.94
Unknown	0.62 (0.47-0.81)	.001	0.99 (0.72-1.34)	.92
Ethnicity				
Hispanic	0.55 (0.45-0.68)	<.001	1.04 (0.82-1.31)	.76
Marital status				
Married	1.04 (0.93-1.17)	.48	0.88 (0.77-1.00)	.06
Comorbid conditions				
Hypertension	3.71 (3.22-4.27)	<.001	1.01 (0.85-1.20)	.89
Ischemic heart disease, cardiac disease intervention, or myocardial infarction	3.28 (2.90-3.71)	<.001	1.12 (0.96-1.30)	.14
Atrial fibrillation	3.52 (3.04-4.07)	<.001	1.11 (0.93-1.32)	.25
Stroke	2.53 (1.98-3.22)	<.001	0.98 (0.75-1.29)	.91
Diabetes or insulin use	2.76 (2.46-3.10)	<.001	1.40 (1.22-1.60)	<.001
Congestive heart failure	3.54 (3.07-4.09)	<.001	0.94 (0.78-1.13)	.50
Chronic kidney disease	3.96 (3.53-4.45)	<.001	1.82 (1.60-2.08)	<.001
Dialysis	3.72 (2.82-4.91)	<.001	1.18 (0.86-1.63)	.30
COPD or bronchiectasis	3.01 (2.63-3.44)	<.001	1.17 (0.99-1.39)	.06
Asthma	0.78 (0.59-1.04)	.09	0.88 (0.65-1.19)	.41
Pneumonia	3.32 (2.71-4.09)	<.001	1.18 (0.93-1.50)	.18
Sleep apnea	1.21 (1.05-1.39)	.009	1.10 (0.93-1.30)	.27
Deep venous thrombus or pulmonary embolism	2.00 (1.51-2.64)	<.001	1.07 (0.79-1.46)	.65
Rheumatoid arthritis or other inflammatory conditions	1.54 (1.11-2.14)	.01	1.16 (0.81-1.67)	.42
Cancer, including prostate cancer	2.42 (2.06-2.84)	<.001	1.07 (0.89-1.27)	.48
HIV and AIDS	0.95 (0.55-1.62)	.84	1.03 (0.58-1.83)	.92
Dementia	7.06 (6.01-8.29)	<.001	2.08 (1.73-2.51)	<.001
Cirrhosis or hepatitis	1.51 (1.25-1.82)	<.001	1.30 (1.05-1.61)	.02
Home oxygen in past year	3.17 (2.55-3.94)	<.001	1.38 (1.07-1.79)	.01
BMI				
<18.5	2.88 (1.65-5.05)	<.001	1.16 (0.62-2.18)	.65
18.5-24.9	1 [Reference]		1 [Reference]	
25.0-29.9	0.75 (0.67-0.84)	<.001	0.83 (0.68-1.00)	.05
≥30	1.72 (1.48-1.99)	<.001	1.01 (0.83-1.22)	.95
Unknown	1.01 (0.82-1.24)	.96	1.01 (0.77-1.32)	.95
Month of positive COVID-19 test result				
March	1 [Reference]		1 [Reference]	
April	2.67 (2.33-3.07)	<.001	0.62 (0.50-0.77)	<.001
May	1.41 (1.15-1.73)	.001	0.35 (0.26-0.46)	<.001
June	0.74 (0.62-0.88)	.001	0.33 (0.25-0.42)	<.001
July	0.48 (0.41-0.55)	<.001	0.23 (0.18-0.29)	<.001
August	0.69 (0.58-0.82)	<.001	0.23 (0.18-0.30)	<.001
September	0.71 (0.59-0.85)	<.001	0.23 (0.18-0.30)	<.001

Abbreviations: BMI, body mass index (calculated as weight in kilograms divided by height in meters squared); COPD, chronic obstructive pulmonary disease; OR, odds ratio.

^a^ Adjustment included age, sex, race, ethnicity, marital status, clinical factors, health care facility, and month of COVID-19 diagnosis.

Housing problems and substance use were higher in Black (1583 [16.2%] and 1482 [15.2%]) and American Indian or Alaska Native (46 [16.3%] and 50 [17.7%]) veterans compared with other racial/ethnic groups (eTable 3 in the Supplement). In contrast, Hispanic veterans had a lower prevalence of housing problems, financial hardship, current tobacco use, and substance use than non-Hispanic White veterans (eTable 4 in the Supplement). Given that Asian and American Indian or Alaska Native races were associated with mortality, we assessed these groups and found no evidence of an interaction with social and behavioral risk factors among these groups (eTable 5 in the Supplement).

## Discussion

Despite relatively high levels of social and behavioral risk factors, no association with mortality from COVID-19 was found among individuals who obtained care through the VA, the largest integrated health care system in the US. Instead, we identified factors associated with mortality that were consistent with those reported in other studies, including older age, Asian and American Indian or Alaska or Alaska Native race, and certain comorbid conditions, such as diabetes and chronic kidney disease. Studies conducted outside of the VA health system have found that social risk factors were associated with mortality.^37,38,39^ Asking why we did not observe this association in the VA allows us to consider how the VA health system may be different from other US health care systems.

The VA has a portfolio of social and behavioral support programs that address housing instability, financial constraints, and substance use disorder.^40,41^ The US Department of Housing and Urban Development–VA Supportive Housing Program, for example, combines housing vouchers and supportive services to help veterans who are homeless and their families to find and sustain permanent housing.^42^ Case managers connect veterans with social and behavioral risk factors to other support services, including mental health treatment and substance use counseling. These services reduce barriers to care.^43,44,45,46^ The strong social and behavioral support programs in the VA may explain why we did not observe an association between social and behavioral risk factors and mortality. Studies that are designed to assess the potential reasons for this finding are needed.

In the general population of the US, concerns are widespread that social and behavioral risk factors may be associated with a much greater burden of disease and increased deaths from COVID-19, particularly among individuals with health disparities.^13^ Yet, we did not find a disproportionate association between these risk factors and mortality among a national cohort of socially vulnerable White, Black, or Hispanic veterans. Instead, the mitigating role may be attributed to the structural equities of the VA system. Further research is needed to substantiate the potential of an integrated system to be a model of support services to households with COVID-19 and populations who are at risk for this disease.

### Limitations

This study has several limitations. First, veteran characteristics and access to support programs and integrated health care differ from those of the general population; thus, these findings should be extrapolated with caution. Second, veterans with poor health behaviors, such as heavy tobacco use, may have died before the pandemic from non–COVID-19 causes and thus were not included in the study, and those who were alive may have been less susceptible to the poor outcomes of their health behaviors; this situation presented the potential for selection bias (also called survivor bias). Third, social risk factors tend to be undercoded *ICD-10* variables, which can lead to measurement error but not bias.^47^ However, we reviewed a random sample of medical records to confirm the classification of the measurements. In addition, the consistency of other findings from covariates, such as age, was reassuring, and we created iterative models to assess the association of age and other covariates with mortality. Fourth, although a large group of covariates were analyzed, certain covariates, such as occupational history, were not available from the VA data and may account for unmeasured confounding. Fifth, this study was not designed to explore the potential reasons for the observed findings.

## Conclusions

This cohort study highlights the lack of association between death from COVID-19 and social and behavioral risk factors in an integrated VA health system. The VA health care system is known to transcend social vulnerabilities and has the potential to serve as a model of support services for households with COVID-19 and populations who are at risk for this disease in the US.

## Notes

Supplement. **eTable 1.** Codes Used to Define the Variables for the Social and Behavioral Risk Factors **eTable 2.** Iterative Prediction Models to Assess Social and Behavioral Risk With 30-Day Mortality Among Veterans With COVID-19 **eTable 3.** Social and Behavioral Risk With COVID-19 Mortality, by Race **eTable 4.** Social and Behavioral Risk With Mortality, by Ethnicity **eTable 5.** Assessment of Interaction Between Social and Behavioral Risk and Race/Ethnicity
