# Supplementary material for: Association of Social and Behavioral Risk Factors With Mortality Among US Veterans With COVID-19
Source: JAMA Netw Open. 2021 Jun 9;4(6):e2113031. doi: 10.1001/jamanetworkopen.2021.13031 (PMC8190626; doi:10.1001/jamanetworkopen.2021.13031)
Supplement: Supplement. — eTable 1. Codes Used to Define the Variables for the Social and Behavioral Risk Factors eTable 2. Iterative Prediction Models to Assess Social and Behavioral Risk With 30-Day Mortality Among Veterans With COVID-19 eTable 3. Social and Behavioral Risk With COVID-19 Mortality, by Race eTable 4. Social and Behavioral Risk With Mortality, by Ethnicity eTable 5. Assessment of Interaction Between Social and Behavioral Risk and Race/Ethnicity [file jamanetwopen-e2113031-s001.pdf]

Supplementary Online Content

Kelly JD, Bravata DM, Bent S, et al. Association of social and behavioral risk factors with mortality among US veterans with COVID-19. *JAMA Netw Open*. 2021;4(6):e2113031. doi:10.1001/jamanetworkopen.2021.13031

eTable 1. Codes Used to Define the Variables for the Social and Behavioral Risk Factors

eTable 2. Iterative Prediction Models to Assess Social and Behavioral Risk With 30-Day Mortality Among Veterans With COVID-19

eTable 3. Social and Behavioral Risk With COVID-19 Mortality, by Race

eTable 4. Social and Behavioral Risk With Mortality, by Ethnicity

eTable 5. Assessment of Interaction Between Social and Behavioral Risk and Race/Ethnicity

This supplementary material has been provided by the authors to give readers additional information about their work.

© 2021 Kelly JD et al. *JAMA Network Open.*

eTable 1. Codes Used to Define the Variables for the Social and Behavioral Risk Factors

Domain	Definitions
**Social Risk Factors**	
Financial hardship	• ICD-10 codes, at least one code in the past two years<br>○ Z59.4 Lack of adequate food and safe drinking water<br>○ Z59.5 Extreme poverty<br>○ Z59.6 Low income<br>○ Z59.7 Insufficient social insurance and welfare support<br>OR<br>• VA priority Score 5: annual income level that's below the VA adjusted income limits-based zip code, eligible to receive VA pension benefits or eligible for Medicaid programs.
Housing problems	• ICD-10 codes, at least one code in past two years<br>○ Z59.0 Homelessness<br>○ Z59.1 Inadequate housing<br>○ Z59.8 Other problems related to housing and economic circumstances<br>○ Z59.9 Problem related to housing and economic circumstances, unspecified<br>OR<br>• Clinic Stop codes, at least one code in past two years: 507, 522, 528, 529, 530
**Behavioral Risk Factors**	
Tobacco abuse	• ICD-10 codes, at least one code in the past year<br>○ F17.200 Nicotine dependence, unspecified, uncomplicated<br>○ F17.210 Nicotine dependence, cigarettes, uncomplicated<br>○ F17.290 Nicotine dependence, other tobacco product, uncomplicated<br>○ Z72.0 Tobacco use<br>OR<br>• CPT code, at least one code in the past year<br>○ 99406 Smoking and tobacco cessation counseling visit, 3 to 10 minutes

© 2021 Kelly JD et al. *JAMA Network Open.*

	◦ 99407 Smoking and tobacco cessation counseling visit, greater than 10 minutes<br>◦ S9075 Smoking cessation treatment<br>◦ S9453 Smoking cessation classes, non-physician provider<br>◦ G0436 Smoking and tobacco cessation counseling for asymptomatic patient, 3 to 10 minutes<br>◦ G0437 Smoking and tobacco cessation counseling for asymptomatic patient, greater than 10 minutes<br>OR<br>• Clinic stop code, at least one code in the past year: 707, 708<br>OR<br>• Most recent health factor within past year indicates current smoker. Table of smoking-related health factors adapted from table available at: https://medicine.yale.edu/intmed/vacs/<br>•
Alcohol use	• Audit C Score>=3 for female, >=4 for male<br><br>OR<br>• One inpatient ICD10 code or two outpatient ICD10 codes within the past two years:<br>◦ F10: All Alcohol related disorders<br>◦ K70 Alcoholic liver disease<br>▪ K70.0 Alcoholic fatty liver<br>▪ K70.1 Alcoholic hepatitis<br>• K70.10 …… without ascites<br>• K70.11 …… with ascites<br>▪ K70.2 Alcoholic fibrosis and sclerosis of liver<br>▪ K70.3 Alcoholic cirrhosis of liver<br>• K70.30 …… without ascites<br>• K70.31 …… with ascites<br>▪ K70.4 Alcoholic hepatic failure<br>• K70.40 …… without coma<br>• K70.41 …… with coma<br>▪ K70.9 Alcoholic liver disease, unspecified<br>◦ G31.2 Degeneration of nervous system due to alcohol<br>◦ G62.1, Alcoholic Polyneuropathy<br>◦ I42.6 Alcoholic Cardiomyopathy<br>◦ K29.2 Alcoholic Gastritis<br>◦ O35.4 All maternal care for (suspected) damage to fetus from alcohol<br>◦ O99.31 All alcohol use complicating pregnancy, childbirth, and the puerperium

© 2021 Kelly JD et al. *JAMA Network Open.*

Substance use	• Any single ICD10 code associated with drug use in an inpatient setting or two ICD10 codes associated with drug use in an outpatient setting within 2 years.<br>○ F11 Opioid related disorders<br>○ F12 Cannabis related disorders<br>○ F13 Sedative, hypnotic, or anxiolytic related disorders<br>○ F14 Cocaine related disorders<br>○ F15 Other stimulant related disorders<br>○ F16 Hallucinogen related disorders<br>○ F17 Nicotine dependence<br>○ F18 Inhalant related disorders<br>○ F19 Other psychoactive substance related disorders<br>○ O35.5 Maternal care for (suspected) damage to fetus by drugs<br>○ O99.32 Drug use complicating pregnancy, childbirth, and the puerperium<br>○ T40 Poisoning by, adverse effect of and underdosing of narcotics and pschodysleptics<br>○ T41.0 Poisoning by, adverse effect of and underdosing of inhaled anesthetics<br>○ T42.6 Poisoning by, adverse effect of and underdosing of other antiepileptic and sedative-hypnotic drugs<br>○ T42.7 Poisoning by, adverse effect of and underdosing of unspecified antiepileptic, and sedative-hypnotic drugs<br>○ T43.6 Poisoning by, adverse effect of, and underdosing of psychostimulants

© 2021 Kelly JD et al. *JAMA Network Open.*

eTable 2. Iterative Prediction Models to Assess Social and Behavioral Risk With 30-Day Mortality Among Veterans With COVID-19

	Crude OR (95% CI)	p	OR adjusted for age (95% CI)	p	OR adjusted for demographics, Charlson comorbidity index, location, month, and all social and behavioral risk factors (95% CI)	p
Housing problems	0.86 (0.71, 1.04)	0.13	1.24 (1.01, 1.51)	0.04	0.98 (0.79, 1.22)	0.88
Financial hardship	1.86 (1.63, 2.12)	< .001	1.18 (1.025, 1.36)	0.02	1.11 (0.96, 1.29)	0.17
Current tobacco use	0.63 (0.53, 0.75)	< .001	0.92 (0.77, 1.1)	0.37	0.86 (0.70, 1.07)	0.17
Alcohol use	0.53 (0.44, 0.63)	< .001	0.76 (0.63, 0.91)	0.004	0.79 (0.65, 0.96)	0.02
Substance use	0.83 (0.69, 0.99)	0.04	1.05 (0.87, 1.27)	0.61	0.92 (0.73, 1.16)	0.48

© 2021 Kelly JD et al. *JAMA Network Open.*

eTable 3. Social and Behavioral Risk With COVID-19 Mortality, by Race

	All (n=27,640)	White (n=15,113)	Black (n=9,745)	Asian (n=328)	American Indian or Alaska Native (n=283)	Native Hawaiian or other Pacific Islander (n=316)	Unknown (n=1,855)	p
Housing problems	3090 (11.2%)	1234 (8.2%)	1583 (16.2%)	27 (8.2%)	46 (16.3%)	30 (9.5%)	170 (9.2%)	< 0.001
Financial hardship	4450 (16.1%)	2403 (15.9%)	1667 (17.1%)	35 (10.7%)	52 (18.4%)	44 (13.9%)	249 (13.4%)	< 0.001
Current smoker	4910 (17.8%)	2665 (17.6%)	1830 (18.8%)	60 (18.3%)	64 (22.6%)	53 (16.8%)	238 (12.8%)	< 0.001
Alcohol use	5358 (19.4%)	2957 (19.6%)	1841 (18.9%)	74 (22.6%)	70 (24.7%)	49 (15.5%)	367 (19.8%)	0.03
Substance use	3569 (12.9%)	1793 (11.9%)	1482 (15.2%)	30 (9.1%)	50 (17.7%)	41 (13%)	173 (9.3%)	< 0.001

© 2021 Kelly JD et al. *JAMA Network Open.*

eTable 4. Social and Behavioral Risk With Mortality, by Ethnicity

	All	Hispanic	Not Hispanic	p
Housing problems	3090 (11.2%)	383 (9.8%)	2707 (11.4%)	0.004
Financial hardship	4450 (16.1%)	540 (13.9%)	3910 (16.5%)	< 0.001
Current smoker	4910 (17.8%)	539 (13.8%)	4371 (18.4%)	< 0.001
Alcohol use	5358 (19.4%)	838 (21.5%)	4520 (19%)	< 0.001
Substance use	3569 (12.9%)	362 (9.3%)	3207 (13.5%)	< 0.001

© 2021 Kelly JD et al. *JAMA Network Open.*

eTable 5. Assessment of Interaction Between Social and Behavioral Risk and Race/Ethnicity

	Housing problems	p	Financial hardship	p	Current smoker	p	Alcohol use	p	Substance use	p
American Indian or Alaska Native and risk factor present	2.78 (0.76, 8.9)	0.10	0.52 (0.13, 1.67)	0.3	0.61 (0.09, 2.45)	0.54	1.14 (0.16, 4.68)	0.87	2.44 (0.65, 7.98)	0.16
Asian and risk factor present	**-	-	0.38 (0.02, 2.37)	0.39	-	-	0.93 (0.05, 5.47)	0.94	-	-

* Model adjusted for age, sex, race, social and behavioral risk, Charlson comorbidity score, and interaction between race and risk covariates.

** Interaction between Asian race and housing problems, tobacco use, and Substance abuse was not assessed due to no deaths among Asian patients with these risk variables.

© 2021 Kelly JD et al. *JAMA Network Open.*
